# Active exercise therapy improves the recovery of knee joint function and reduction of muscle atrophy after medial patellofemoral ligament reconstruction for recurrent patellar dislocation

**DOI:** 10.3389/fsurg.2022.954287

**Published:** 2022-11-01

**Authors:** Dong Xing, Wenyi Li, Zhaoxu Yang, Zhijie Dong, Huijun Kang, Fei Wang

**Affiliations:** ^1^ Department of Joint Surgery, Third Hospital of Hebei Medical University, Shijiazhuang, China; ^2^ Department of Orthopedics, Hebei General Hospital, Shijiazhuang, China

**Keywords:** recurrent patellar dislocation, medial patellofemoral ligament reconstruction, postoperative exercise therapy, kujala score, lysholm score, muscle atrophy

## Abstract

**Objectives:**

Medial patellofemoral ligament (MPFL) reconstruction is an important surgical therapy for recurrent patellar dislocation. However, few studies have focused on exercise therapy after MPFL reconstruction. Therefore, the first purpose was to compare the active and traditional postoperative exercise therapies on the recovery of knee joint function and reduction of muscle atrophy after MPFL reconstruction, and the second purpose was to compare the active and traditional postoperative exercise therapies on the patellar stability after MPFL reconstruction.

**Methods:**

The cases of 31 patients with recurrent patellar dislocation treated with patella double semi-tunnel anatomical MPFL reconstruction from February 2016 and February 2019 were retrospectively reviewed. The clinical outcomes, including the patellar tilt angle (PTA), lateral patellofemoral angle (LPFA), thigh circumference reduction, Kujala score, and Lysholm score, were compared between two groups (i.e., active exercise and traditional exercise groups) preoperatively, 3 months postoperatively, 6 months postoperatively, 12 months postoperatively, and 24 months postoperatively.

**Results:**

The Kujala score was significantly higher in the active exercise group than traditional exercise group 3 months postoperatively (80.06 vs. 74.80, *P* < 0.01), 6 months postoperatively (89.19 vs. 82.07, *P* < 0.01), 12 months postoperatively (91.43 vs. 86.60, *P* < 0.01), and 24 months postoperatively (92.50 vs. 90.27, *P* = 0.02). Similarly, there was a higher Lysholm score in the active exercise group compared with traditional exercise group 3 months postoperatively (81.25 vs. 76.53, *P* < 0.01), 6 months postoperatively (89.81 vs. 84.80, *P* < 0.01), 12 months postoperatively (93.25 vs. 88.40, *P* < 0.01), and 24 months postoperatively (93.69 vs. 90.67, *P* < 0.01). Significantly lower thigh circumference reduction was reported in the active exercise group compared with that in the traditional exercise group 3 months postoperatively (1.90 ± 0.57 vs. 2.45 ± 0.45, *P* < 0.01) and 6 months postoperatively (1.50 ± 0.31 vs. 1.83 ± 0.32, *P* < 0.01). No statistical difference was observed between the two groups in terms of PTA (*P* > 0.05) or LPFA postoperatively (*P* > 0.05).

**Conclusions:**

Our results suggested that active exercise therapy might benefit the early recovery of knee joint function and reduction of muscle atrophy in patients with recurrent patellar dislocation after MPFL reconstruction.

## Introduction

Patellar dislocation is a common knee injury, accounting for 2% to 3% of all knee injuries worldwide (1, 2). Recurrent dislocation can occur in 17% to 45% of patients due to the patellar instability after the initial dislocation, and surgical treatment is routinely recommended (2–5). The medial patellofemoral ligament (MPFL) is the primary soft tissue stabilizer for preventing the lateral dislocation of patella. However, the MPFL typically ruptures in cases of patellar dislocation (6, 7). The double-bundle anatomical reconstruction of the MPFL is an important surgical therapy for recurrent patellar dislocation to achieve the satisfactory biomechanical function of the MPFL and the patellofemoral joint (8, 9).

An appropriate exercise therapy after MPFL reconstruction is as important as the surgical process in the treatment of recurrent patellar dislocation given its vital effect on the recovery of the knee joint function; however, there is no consensus on the postoperative exercise therapy recommend (10). Several studies have reported that exercise therapy should start 1–2 weeks after surgery, and the flexion angle of the knee could reach 90° at 4 weeks postoperatively (10–12). In Wang et al. study, flexion of 10° was allowed from the 3 day after surgery and the angle was increased gradually to 90° in a month after MPFL reconstruction (13). Zhou et al. study indicated that 6 weeks of exercise were necessary for the flexion angle of the knee to reach 90° (14). Other studies showed that the flexion angle of the knee could reach 90° at 2 weeks postoperatively (15, 16). Generally, joint adhesion might exist if the flexion angle of the knee remained <90° for a long time, but the compulsory functional exercise might lead to patella fracture (17). To our knowledge, studies that compare the effects of different exercise types on the recovery of the knee joint function and reduction of muscle atrophy remained limited despite the considerable importance of postoperative exercise in recurrent patellar dislocation.

Therefore, this study was retrospectively performed on patients with recurrent patellar dislocation treated with MPFL reconstruction. The first purpose of this study was to compare the active and traditional postoperative exercise therapies on the recovery of knee joint function and reduction of muscle atrophy after MPFL reconstruction, and the second purpose was to compare the active and traditional postoperative exercise therapies on the patellar stability after MPFL reconstruction.

## Materials and methods

### Patient population

This study was approved by the ethics committee of our hospital, and informed consent was obtained from each patient included in the research. The cases of patients with recurrent patellar dislocation who had undergone patella double semi-tunnel anatomical MPFL reconstruction between February 2016 and February 2019 were retrospectively reviewed.

The inclusion criteria were as follows: (1) patellar dislocation occurred at least two times; (2) patellar instability following the initial dislocation persisted for more than three months; (3) magnetic resonance imaging indicated the injury, and even rupture, of the MPFL; (4) grade 1 or 2 focal articular cartilage defects according to the Outerbridge standard (18); (5) patients did not receive surgical treatments for the patellar dislocation before this research, and (6) DeJour classification of trochlear morphology ≤ grade B (19). Patients with the following conditions were excluded from the study: (1) undergone previous surgeries on an injured knee; (2) *Q* angle >20° (20); (3) trochlear angle >145°; (4) tibial tuberosity–trochlear groove distance >20 mm; (5) grades IV and V patellar dysplasia (Wiberg classification) (21); (6) patella alta (Insall–Salvati index >1.2); (7) grade 3 or 4 focal articular cartilage defects according to the Outerbridge standard (18), and (8) bilateral patellar dislocations. Specially, DeJour classification system was constructed based on the combined evaluation of axial and lateral radiographs. Type A dysplasia is characterized by a crossing sign on the lateral view and a sulcus angle >145° on the axial view. Type B dysplasia is characterized by the sign of a flat trochlea on axial views and a supratrochlear spur on lateral views. Type C dysplasia is characterized by the appearances of a crossing sign and a double contour on lateral radiographs as well as the medial hypoplasia and lateral convexity on axial radiographs. Type D dysplasia is characterized by asymmetry of the trochlear facets and a cliff between the medial and lateral facets on the axial radiographic view (19).

### Surgical technique

**Figure 1:**
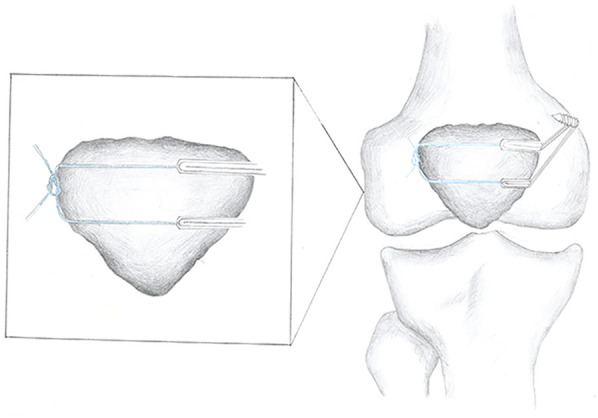
Schematic diagram for MPFL reconstruction of patella double semi-tunnel.

The details of the surgical technique were described in our previous studies (22, 23). The semitendinosus tendon was harvested by a closed-end tendon stripper, and the tendon was divided longitudinally into two grafts. A double rings-shaped graft was made by folding the midpoint of the two grafts. The four free ends were interlacing sutures with a length of 25 mm as the femoral end and the two rings were used as the patella end. The MPFL was fastened at the posterosuperior part of the medial condyle of the femur and the center and medial patellar margins of the patella in 30° of knee flexion (Figure 1).

### Postoperative exercise

**Figure 2:**
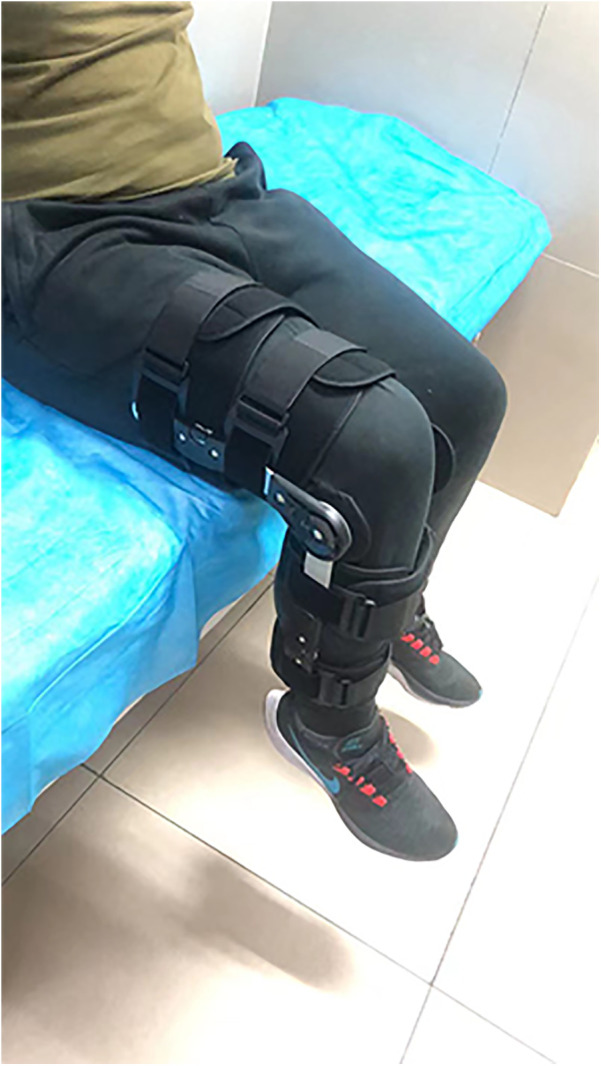
The adjustable brace used in the postoperative exercise.

All the patients were divided into the active exercise group and the traditional exercise group on the basis of the training schedule of the knee flexion. In the active exercise group, patients were required to start the knee flexion exercise 5 days postoperatively with the assistance of an adjustable brace (Dagao, Shanghai, China) (Figure 2). The degree of knee flexion increased by approximately 10° every day until 90° was reached within 2 weeks. Then, the degree of knee flexion increased by 10° every 5 days until 120° was reached within 4 weeks. The support would be removed, and the knee exercise would not be limited to 6 weeks postoperatively. In the traditional exercise group, patients were required to start knee flexion exercise 1 week postoperatively with the assistance of an adjustable brace. The degree of knee flexion increased by 30° every week until 90° was reached within 4 weeks. Then, the degree of knee flexion increased by 10° every 5 days until 120° was reached within 6 weeks. The support would be removed, and the knee exercise would not be limited to 8 weeks postoperatively (24). Moreover, it should be noted that previous studies have shown that open-chain and closed-chain exercises were used to train isolated muscle groups and the functional integration of multiple muscle groups of the lower extremity, respectively. The outcome of closed-chain exercise after MPFL reconstruction was superior to that of open-chain exercise (25, 26). Therefore, we combined open-chain and closed-chain exercises in the current study to improve muscle strength.

With respect to weight training, all patients began to gradually bear the body weight with the assistance of a brace 2 weeks postoperatively, and they would be permitted to bear full body weight at 4 weeks postoperatively. For strength training, ankle pump exercises were used to promote blood circulation and prevent thrombosis after surgery. The straight leg raise exercises started at the third day postoperatively, and the half-squat exercises started at 6 weeks postoperatively, and both of them stopped at 12 weeks postoperatively. Moreover, all the patients started the patella-shifting exercise by shifting the patella medially at 3 days postoperatively until the knee joint reached its full range of motion (ROM) at 8 weeks postoperatively to reduce the tension of the reconstructed MPFL and promote the healing of soft tissues.

All patients could walk normally without the support of a brace at 8 weeks postoperatively. Patients could perform short-distance accelerated running and long-distance slow running at 12 weeks postoperatively. They could do normal physical exercise, such as running and rotational motion of the knee, at 26 weeks postoperatively.

### Clinical outcomes

**Figure 3:**
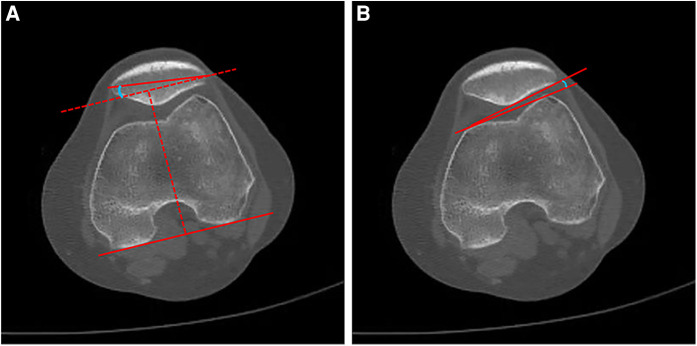
Schematic diagrams for PTA (**A**) and LPFA (**B**).

The ROM of the knee was measured using a universal goniometer. Thigh circumference was measured 5 cm above the patella superior border by using a medical ruler (26). Thigh circumference reduction was calculated by subtracting the preoperative data from the postoperative data. The patellar tilt angle (PTA) and the lateral patellofemoral angle (LPFA) were measured using the computed tomography data of the knee at 30° flexion. The PTA referred to the angle between the mediolateral patellar axis and a line drawn through the posterior condyles at the deepest part of the sulcus (Figure 3A), and the LPFA was defined as the acute angle between a line joining the anterior margins of both femoral condyles and the lateral patellar facet (Figure 3B). The ROM of the knee, thigh circumference reduction, PTA, and LPFA were measured three times, and the average value was considered the final result. The function of the knee joint was evaluated through the Kujala score and the Lysholm score (27, 28). Specifically, the Kujala score was composed of thirteen subscales with a score ranging from 5 to 10 points for each subscale. The Kujala score has been widely used to evaluate subjective symptoms and functional limitations of patellofemoral disorders (27). The Lysholm score was used to evaluate the knee joint function from eight following items: claudication, squatting, support, up stairs, pain, instability, sense of closure and swelling. A higher Lysholm score indicated better knee function, and the total score was 100 points (28). Major complications, such as recurrent dislocation or fracture of the patella, were recorded during the follow-up. All clinical data were collected preoperatively, 3 months postoperatively, 6 months postoperatively, 12 months postoperatively, and 24 months postoperatively.

### Statistical analysis

The independent sample *t*-test or Mann-Whitney *U* test was used for the continuous variables, and the Fisher exact test was used for the categorical variables to determine the difference between the two groups. The paired sample *t*-test or Wilcoxon signed rank test was used to detect changes during the treatment for continuous variables for the same one patient. Normally distributed data were represented by mean ± standard deviation (SD), and non-normally distributed data were represented by a median with a range. A statistical difference was indicated by *P* < 0.05. All the analyses were performed using SPSS 20.0 (SPSS Inc., USA).

## Results

### General information

A total of 36 patients underwent MPFL reconstruction for recurrent patellar dislocation in our hospital from February 2016 and February 2019. Among them, 5 patients were excluded from this study because of the following reasons: bilateral patellar dislocation (*n* = 2), lost to the follow-up (*n* = 2), and insufficient data (*n* = 1). Finally, 31 patients were included in this study.

The demographic data of all the patients are provided in Table 1. The patients included 13 males and 18 females, with a median age of 18 years (range: 15–53) and a mean body mass index (BMI) of 22.60 ± 3.15 kg/m^2^. The median follow-up time was 30 months (range: 25–48 months). The active exercise group comprised 16 patients, while the conventional exercise group consisted of 15 patients. No significant difference was observed between two groups in terms of gender (*P* = 1.00), age (*P* = 0.90), surgical side (*P* = 0.72), BMI (*P* = 0.58), interval between the first dislocation and the surgery (*P* = 0.35), or follow-up time (*P* = 0.07). All patients completed the exercise plan, and there was no complication relevant to the exercise therapy during the regular follow-up, including recurrent dislocation and patellar instability.

**Table 1:** Demographics of included patients.

Variables	Total (*n* = 31)	Active exercise group (*n* = 16)	Traditional exercise group (*n* = 15)	*P*
Gender (male/female)	13/18	7/9	6/9	1.00
Age (median, range) (year)	18 (15–53)	17.5 (15–51)	18 (15–53)	0.90
Surgical site (left/right)	15/16	7/9	8/7	0.72
BMI (mean ± SD) (kg/m^2^)	22.60 ± 3.15	22.28 ± 3.17	22.92 ± 3.20	0.58
Interval between the first dislocation and recurrent dislocation (median, range) (month)	7.00 (2–17)	6.50 (2–16.5)	7.00 (5–17)	0.35
Follow-up time (median, range) (month)	30 (25–48)	33 (25–48)	28 (25–46)	0.07

BMI, body mass index; SD, standard deviation.

The details of clinical outcomes are provided in Table 2. No significant difference was found between the two groups with respect to Kujala score, Lysholm score, thigh circumference, PTA, and LPFA preoperatively (*P* > 0.05). Kujala score, Lysholm score, PTA, and LPFA improved considerably postoperatively compared with preoperatively (*P* < 0.05). Patients in the active exercise group had significantly higher Kujala score compared with those in the traditional exercise group 3 months postoperatively (80.06 vs. 74.80, *P* < 0.01), 6 months postoperatively (89.19 vs. 82.07, *P* < 0.01), 12 months postoperatively (91.43 vs. 86.60, *P* < 0.01), and 24 months postoperatively (92.50 vs. 90.27, *P* = 0.02). Similarly, patients in the active exercise group had significantly higher Lysholm score compared with those in the traditional exercise group 3 months postoperatively (81.25 vs. 76.53, *P* < 0.01), 6 months postoperatively (89.81 vs. 84.80, *P* < 0.01), 12 months postoperatively (93.25 vs. 88.40, *P* < 0.01), and 24 months postoperatively (93.69 vs. 90.67, *P* < 0.01). Moreover, the thigh circumference reduction in the active exercise group was significantly lower than that in the traditional exercise group 3 months postoperatively (1.90 vs. 2.45, *P* < 0.01) and 6 months postoperatively (1.50 vs. 1.83, *P* < 0.01), but the significant difference disappeared 12 months postoperatively (*P* = 0.32) and 24 months postoperatively (*P* = 0.20). However, no evident difference was observed between two groups in terms of PTA (*P* > 0.05) or LPFA (*P* > 0.05) postoperatively.

**Table 2:** Comparison of clinical outcomes between two groups.

Variables	Time point	Active exercise group (*n* = 16)	Traditional exercise group (*n* = 15)	*P*
Kujala score	Preoperatively	50.12 ± 5.84	51.33 ± 5.18	0.55
	3 months postoperatively	80.06 ± 2.21**	74.80 ± 2.04**	<0.01*
	6 months postoperatively	89.19 ± 2.37**	82.07 ± 2.40**	<0.01*
	12 months postoperatively	91.43 ± 2.92**	86.60 ± 2.77**	<0.01*
	24 months postoperatively	92.50 ± 2.48**	90.27 ± 2.66**	0.02*
Lysholm score	Preoperatively	50.75 ± 5.46	49.93 ± 5.39	0.68
	3 months postoperatively	81.25 ± 3.45**	76.53 ± 4.63**	<0.01*
	6 months postoperatively	89.81 ± 3.45**	84.80 ± 3.51**	<0.01*
	12 months postoperatively	93.25 ± 2.86**	88.40 ± 3.20**	<0.01*
	24 months postoperatively	93.69 ± 2.47**	90.67 ± 2.79**	<0.01*
Thigh-circumference reduction (cm)	Preoperatively (thigh- circumference)	38.57 ± 2.44	39.27 ± 2.21	0.41
	3 months postoperatively	1.90 ± 0.57	2.45 ± 0.45	<0.01*
	6 months postoperatively	1.50 ± 0.31	1.83 ± 0.32	<0.01*
	12 months postoperatively	1.20 ± 0.17	1.26 ± 0.17	0.32
	24 months postoperatively	0.47 ± 0.17	0.55 ± 0.19	0.20
PTA (degree)	Preoperatively	26.31 ± 3.82	25.00 ± 4.00	0.36
	3 months postoperatively	9.88 ± 2.13**	9.40 ± 2.06**	0.53
	6 months postoperatively	11.00 ± 2.03**	11.33 ± 1.63**	0.62
	12 months postoperatively	12.12 ± 1.86**	12.40 ± 1.76**	0.68
	24 months postoperatively	12.94 ± 1.84**	13.47 ± 2.10**	0.46
LPFA (degree)	Preoperatively	−9.50 ± 3.14	−8.87 ± 2.95	0.57
	3 months postoperatively	8.50 ± 1.50**	9.33 ± 1.50**	0.13
	6 months postoperatively	6.63 ± 1.09**	7.20 ± 1.21**	0.17
	12 months postoperatively	5.94 ± 0.86**	6.07 ± 0.96**	0.70
	24 months postoperatively	5.81 ± 0.83**	6.00 ± 0.65**	0.49

PTA, patellar tilt angle; LPFA, lateral patellofemoral angle.

* *P* < 0.05 indicating the significant difference between two groups.

** *P* < 0.05 indicating the significant difference between preoperatively and postoperatively.

## Discussion

Although different rehabilitation plans for improving the knee function after MPFL reconstruction have been proposed (29, 30), few reports focused on the effect of active postoperative exercise on the recovery of knee joint function and patellar stability after MPFL reconstruction. In our study, for the first time, we determined that active postoperative exercise was beneficial for the early recovery of knee joint function and reduction of muscle atrophy without the adverse effect on the patellar stability after MPFL reconstruction.

In the current study, we used the traditional tunnel bioabsorbable interference screw to fix the femoral end of the ligament (31). Similar to our previous report (32), the Kujala score and Lysholm score were significantly improved after the surgery in the current study, and PTA and LPFA were evidently corrected in all patients. Moreover, no complication (e.g., patella fracture or patellar pain) was reported in our study. Therefore, MPFL reconstruction was effective and safe for the surgical management of recurrent patellar dislocation.

Postoperative rehabilitation exercise plays a crucial role in the recovery of the knee joint function after MPFL reconstruction, however, no consensus exists regarding the exercise schedule (16, 17). On the one hand, an excessively aggressive postoperative exercise may disturb the healing of the tendon–bone contact area and adjacent soft tissues; on the other hand, long-time immobilization and an excessively passive postoperative exercise of the surgical knee joint may result in many relevant complications, such as joint adhesion (16, 17). In general, 2–4 weeks postoperatively have been considered the key period for the reconstructed ligament and should be fully utilized to begin postoperative exercise (33). Several studies have provided the theoretical basis of the benefits and safety of early exercise after MPFL rehabilitation. Zhang et al. showed that the tibia will internally rotate and then the patella would move slightly inward to ensure the balance of the patellofemoral joint during the flexion of the knee joint from 0° to 30° (34). The MPFL was tighter during the knee extension than the knee flexion; therefore, the ligament structure would not be significantly stretched during the early rehabilitation exercise (35). Moreover, the fixation of the final ligament during the surgery was performed at the position of 30° flexion of the knee joint, where the MPFL had the most evident force to resist the outward movement of the patella and minimize ligament stretching during postoperative exercise (36). Moreover, compared with the single-bundle MPFL reconstruction, the double-bundle MPFL reconstruction can share the stretching force and provide sufficient protection for early postoperative exercise (37). Similar to the study of Niu et al. (38), no apparent difference in terms of PTA and LPFA was observed between the two groups, indicating that active postoperative exercise was safe and would not affect the patellar stability after MPFL reconstruction.

In recent years, an increasing number of studies have shown that early postoperative exercise helped patients return to their activity level before the injury by alleviating pain, reducing fluid accumulation, strengthening the quadriceps muscle and neuromuscular control, promoting healing, and preventing capsule contracture (16). Ronga et al. found that although the patient had restored previous activities and was satisfied with the operation, a significant drop in the muscle mass of the injured lower extremity was recorded after the rehabilitation exercise (39). Our results showed that the average change in thigh circumference reduction in the active exercise group was significantly lower than that in the traditional exercise group, indicating that faster postoperative exercise could improve the muscle atrophy and recovery of muscle strength in the early and middle periods. In addition, the proprioception of joint would be reduced once patellar dislocation occurred, and the improvement of the Kujala score and Lysholm score of the knee joint after MPFL reconstruction could reflect the recovery of joint proprioception and function (40). In the current study, we discovered that the Kujala score and Lysholm score of the active exercise group were higher than those of the traditional exercise group, indicating that active postoperative exercise could improve the function and proprioception of the knee joint better than traditional exercise. Therefore, patients with recurrent patellar dislocation treated with MPFL reconstruction should practice active postoperative exercise under the guidance of doctors.

Several limitations should be considered when interpreting our results. First, the retrospective design might lead to selection bias, which reduced the accuracy of our results. Second, the sample size in the current study was relatively small, probably decreasing the stringency of the results. Third, follow-up time was relatively short for the assessment of PTA and LPFA, and a longer follow-up period is necessary to confirm our results. Forth, the factor of leg dominance was not considered when calculating the thigh circumference. Notably, we are conducting a prospective study that focuses on this topic with a larger sample size and a longer follow-up period. We believe our future results can eliminate the aforementioned limitations.

## Conclusion

Compared with traditional exercise, active exercise after MPFL reconstruction was beneficial for the early recovery of knee joint function and reduction of muscle atrophy in patients with recurrent patellar dislocation without harmful effects on the patellar stability.

## Data Availability

The raw data supporting the conclusions of this article will be made available by the authors, without undue reservation.
